# Contribution of Resident Memory CD8^+^ T Cells to Protective Immunity against Respiratory Syncytial Virus and Their Impact on Vaccine Design

**DOI:** 10.3390/pathogens8030147

**Published:** 2019-09-11

**Authors:** Angello Retamal-Díaz, Camila Covián, Gaspar A. Pacheco, Angelo T. Castiglione-Matamala, Susan M. Bueno, Pablo A. González, Alexis M. Kalergis

**Affiliations:** 1 Millennium Institute on Immunology and Immunotherapy, Departamento de Genética Molecular y Microbiología, Facultad de Ciencias Biológicas, Pontificia Universidad Católica de Chile, Santiago 8331010, Chile; 2 Departamento de Endocrinología, Escuela de Medicina, Facultad de Medicina, Pontificia Universidad Católica de Chile, Santiago 8331010, Chile

**Keywords:** human respiratory syncytial virus, human orthopneumovirus, respiratory infection, resident memory T cells, vaccine development

## Abstract

Worldwide, human respiratory syncytial virus (RSV) is the most common etiological agent for acute lower respiratory tract infections (ALRI). RSV-ALRI is the major cause of hospital admissions in young children, and it can cause in-hospital deaths in children younger than six months old. Therefore, RSV remains one of the pathogens deemed most important for the generation of a vaccine. On the other hand, the effectiveness of a vaccine depends on the development of immunological memory against the pathogenic agent of interest. This memory is achieved by long-lived memory T cells, based on the establishment of an effective immune response to viral infections when subsequent exposures to the pathogen take place. Memory T cells can be classified into three subsets according to their expression of lymphoid homing receptors: central memory cells (T_CM_), effector memory cells (T_EM_) and resident memory T cells (T_RM_). The latter subset consists of cells that are permanently found in non-lymphoid tissues and are capable of recognizing antigens and mounting an effective immune response at those sites. T_RM_ cells activate both innate and adaptive immune responses, thus establishing a robust and rapid response characterized by the production of large amounts of effector molecules. T_RM_ cells can also recognize antigenically unrelated pathogens and trigger an innate-like alarm with the recruitment of other immune cells. It is noteworthy that this rapid and effective immune response induced by T_RM_ cells make these cells an interesting aim in the design of vaccination strategies in order to establish T_RM_ cell populations to prevent respiratory infectious diseases. Here, we discuss the biogenesis of T_RM_ cells, their contribution to the resolution of respiratory viral infections and the induction of T_RM_ cells, which should be considered for the rational design of new vaccines against RSV.

## 1. Introduction

RSV is the most common responsible pathogen for acute lower respiratory tract infections (ALRI) [1]. RSV causes respiratory distress mostly in young infants, immuno-compromised patients and senior adults [2]. ALRI is the global leader in respiratory tract infections alongside RSV, which causes 45% of young children hospitalizations and in-hospital deaths in infants younger than six months old [3]. Therefore, RSV remains one of the pathogens deemed most important for vaccine development. 

RSV is an enveloped, cytoplasmic virus with a single-stranded, non-segmented, negative-sense RNA genome. This virus belongs to the *Pneumoviridae* family, genus *Orthopneumovirus*, which includes three viral species: *bovine orthopneumovirus*, *murine orthopneumovirus* and *human orthopneumovirus* [4]. The RSV genome is approximately 15.2 kb in size and encodes ten genes in the following order: 3′-NS1-NS2-N-P-M-SH-G-F-M2-L-5′. The M2 gene is transcribed in 2 different ORFs, coding for two proteins, M2-1 and M2-2. The genome is encapsidated by multiple copies of the nucleoprotein (N), forming a helical nucleocapsid [5]. Phosphoprotein (P) copies interact with N and recruit the L protein, an RNA-dependent RNA polymerase and the cofactor M2-1 to the viral nucleocapsid complex. Interestingly, the N protein (a restricted cytosolic protein) can also be expressed on the surface of RSV-infected dendritic cells (DCs). Particularly, the N protein interferes with the interaction of the T cell receptor (TCR) with peptides presented on major histocompatibility complex molecules (pMHC) and inhibits the immunological synapse assembly [6,7]. The RSV genome also encodes for two non-structural proteins, NS-1 and NS-2, which display immunomodulatory properties by inhibiting the induction of interferon-alpha/beta (IFNs α/β) in lung epithelial cells and macrophages [8].

The immune system has the capacity to “remember” pathogens, which is the reason why upon pathogen reencounter, a faster and effective response takes place to establish an adaptive and protective immunological response, which is known as immunological memory [9]. During primary respiratory virus infections, antigen-specific CD8^+^ T cells play fundamental roles in the resolution of infections generated by this type of intracellular pathogens. The adaptive immune response also contributes by conferring protection against subsequent reinfections via immune T and B cell memory development [10]. Thus, frequent reinfections caused by RSV suggest the absence of long-lasting protective immune memory in the host [2,11]. Memory T cells have been classified based on their trafficking patterns and the expression of lymphoid homing receptors CCR7 (chemokine receptor type 7) and CD62L (L-selectin); central memory T cells (T_CM_; CCR7^high^/CD62L^high^) recirculate through the blood and secondary lymphoid organs (SLO), while effector memory T cells (T_EM_; CCR7^low^/CD62L^low^) do not express homing molecules to lymphoid organs, but instead express migratory receptors with the potential of transit through the blood, lymphoid and peripheral tissues (non-lymphoid tissues) [12]. Nonetheless, parabiosis studies have shown that some tissues, namely the gut and brain, are only under immunosurveillance by effector T cells, but not memory T cells, because the circulation of these cells through peripheral tissue is different for each T cell phenotype [13,14,15,16]. The method as to how these tissues are protected was assessed in subsequent studies where a third subset of memory T cells was identified. This subset corresponds to resident memory T cells (T_RM_; CCR7^+^/CD62L^+^) that are permanently present in non-lymphoid tissues and can mediate innate and adaptive immune responses against reinfections with pathogens [17,18,19]. T_RM_ cells constitutively express high levels of the sphingosine-1-phosphate receptor 1 (S1PR1) antagonist CD69 and hyaluronic acid (HA), which binds CD44, collagen-binding CD49a and CD103 (integrin αE paired with integrin chain β7) [18]. Importantly, the role of CD8^+^ T_RM_ cells in tissues has been studied more than that of CD4^+^ T_RM_ lymphocytes. Nevertheless, both cell types participate in the development of the immune response near the locations they occupy and secrete cytokines that promote the recruitment of other immune cells. Several studies have provided evidence for the importance of T_RM_ during protective immune response against RSV [20,21,22,23]. Thus, this response constitutes a critical mechanism that confers immune protection to the host from infections and may also be crucial for the proper function of vaccines, especially for the case of RSV infection.

## 2. Immunological Synapse and Memory T cells

**Figure 1:**
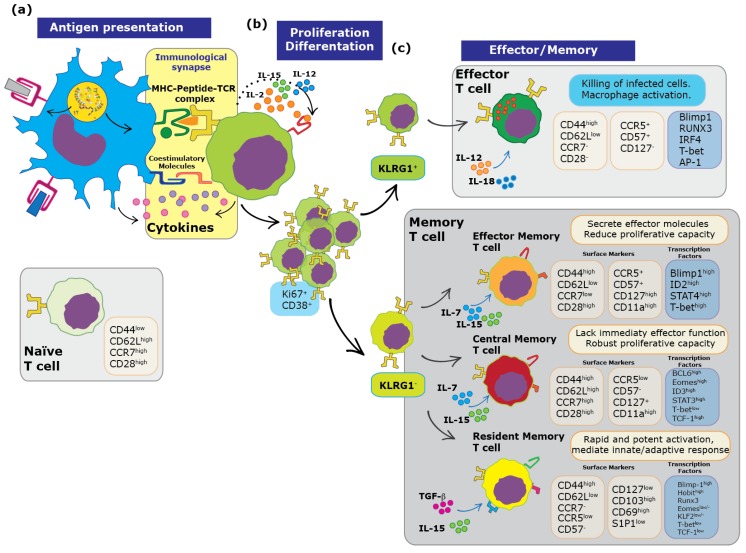
Steps occurring during the activation and differentiation of T lymphocytes. (**a**). Antigen Presentation. Activation of T cells requires the assembly of an immunological synapse with APCs, which provides three types of signals: i) interaction between the TCR and a peptide-loaded MHC class I or class II molecule, ii) co-stimulatory molecule signaling and iii) specific cytokines. (**b**). Proliferation/Differentiation. T cell activation induces T cell proliferation to clonally select and expand antigen-specific T cells. (**c**). Effector functions, surface markers and some cytokines required for homeostasis [53,54,55,56,57,58] and transcription factors that characterize each subpopulation of memory T cells. Central memory T cells (T_CM_) express lymphoid homing markers and circulate through secondary lymphoid organs (SLO). The effector memory T cells (T_EM_) lack expression of lymphoid homing markers, but express other migratory receptors with the potential to migrate through non-lymphoid tissues. The third subset of memory T cells, which consists of resident memory T cells (T_RM_), stably reside in non-lymphoid tissues and contribute to enhancing innate and adaptive immunity against pathogens.

When initiating an immune response, professional antigen presenting cells (APCs), such as DCs, play crucial roles in the intercommunication between innate and adaptive immunity, as well as the modulation of this response [24]. DCs and macrophages are the major APCs in various mucosal tissues, such as the intestine, lung, genital tract and mouth, where they sense and capture microbes and particles that are potentially harmful to the host [25]. Once captured, antigens derived from microbes are processed and presented to naïve T cells at the regional draining lymph nodes [26]. Activation of T cells requires the assembly of an immunological synapse that provides three types of signals: interaction between the TCR and a peptide-loaded MHC class I or class II molecule (pMHC), co-stimulatory molecule signaling and specific cytokines (Figure 1a). If a cell does not receive a full set of signals, it will neither divide nor become activated, but instead may become anergic. The activation of T cells is triggered by TCR-transduced signals upon engagement with a cognate pMHC complex displayed at the cell surface of an APC. Naïve T cells are activated after their encounter with antigens through TCR signaling, inducing clonal expansion. This process is known as priming. Moreover, T cells can differentiate into distinct classes of effector T cells. After pathogen elimination, the existing T cell populations undergo an inflammation-mediated contraction phase, the survivors of which will serve as the initial memory pool [27,28,29]. Furthermore, although the establishment of effective and long-lasting protective immunity has been historically associated with antigen-specific antibody titers, it may also depend on the generation of specific multifunctional effector memory T cells, which are able to eradicate intracellular infections with the pathogen when accompanied by poor or non-effective humoral responses, as seen for hepatitis C virus, tuberculosis, vesicular stomatitis virus, varicella zoster virus, dengue virus and respiratory syncytial virus [6,7,23,30,31,32,33,34,35]. In contrast, the requirement of both antibodies and multifunctional effector memory T cells has been described during infection with malaria [36,37], simian/human immunodeficiency virus [38], *Salmonella* [39] and influenza virus [40,41].

TCR signaling leads to the expression of many genes and the entry of T cells into the cell cycle. There are three significant variables that determine the effects of TCR stimulation in T cells: the affinity and half-life of interaction between the TCR and the pMHC, as well as the phenotype of the APC [6,7,24,42,43,44,45,46]. T cell activation induces T cell proliferation to clonally select and expand antigen-specific T cells (Figure 1b). The transition from naïve T cells into effector cells occurs with changes in cell function. CD8^+^ cytotoxic T cell (CTL) effector functions are stimulated by antigen recognition of a cognate MHC class I–peptide complex by the TCR, inducing apoptosis in the target cell [10]. Since many viruses have developed mechanisms to prevent presentation of their antigens on MHC-I molecules in order to prevent the activation of CD8^+^ T cells, the development, abundance and antiviral effector functions of T_RM_ cells are usually dampened during viral infections [47]. Along these lines, several reports have shown that RSV prevents the development of protective immunological memory by interfering with the capacity of the DCs to activate and elicit effector activities of virus-specific CD4^+^ and CD8^+^ T cells [6,7,48,49]. RSV-infected DCs dampen the activation of naïve CD4^+^ T cells, which downregulate the TCR-driven activation marker CD69 and show reduced secretion of IL-2 [6]. Similarly, immunization of mice with formalin-inactivated RSV (FI-RSV) leads to a pathology known as vaccine-enhanced respiratory disease (ERD), which elicits a pathological immune reaction after vaccination when the individual is exposed to RSV. This disease is characterized by exacerbated eosinophil infiltration and the development of a T_H2_-like response in lungs [50,51]. FI-RSV immunization does not activate a CD8^+^ T cell response that contributes to the eosinophil infiltration in lungs and increased pathology [52]. It is thought that this immune modulatory mechanism could in part be responsible for the lack of generation of long-lasting immunological memory after RSV infection.

## 3. Tissue-Resident Memory T Cells: Biogenesis, Transcription Factors and Surface Markers

Among the cellular components that constitute the first line of defense is a population of memory T cells called tissue-resident memory CD8^+^ T cells (T_RM_). These cells are found in mucosal tissues such as the lungs (both airways and parenchyma), digestive [15] and urogenital tracts [59]. T_RM_ cells have been characterized in elegant parabiosis experiments, which show that tissue surveillance is a function performed by a subset of memory T cells with different characteristics and phenotype behavior than T_EM_ cells, to which this function was previously attributed [59,60]. Besides, once generated, T_RM_ cells can persist at the site of both the infection and vaccination [61]. 

After respiratory virus infection, CD8^+^ T cell priming takes place in the regional draining lymph nodes after lung-resident DCs have transported viral antigens to that site. Interestingly, CD103^+^ DCs have the ability to cross-present foreign antigens to CD8+ T cells, conferring protection against infection [62]. Two migratory DC subsets have been described in the mouse lung tissue, namely CD103^+^-DCs (CD11c^+^/CD11b^low^/CD103^+^ DC) and CD11b^high^-DCs (CD11c^+^/CD11b^high^/CD103^−^). These DC-subsets show differential T cell-stimulatory functions, resulting in a range of distinct memory CD8^+^ T cell subsets [30,63]. CD8^+^ T cells activated by CD103^+^ DCs proliferate vigorously and acquire potent effector properties that, among others, bring them back to the lung to eliminate virus-infected cells. Importantly, subsequent differentiation into T_RM_ cells after influenza virus infection was reported to require priming by CD103^+^ respiratory DCs for the development of resident memory T cell phenotypes [64]. Interestingly, differences in the migration and antigen presentation times between CD103^+^- and CD11b^high^-DCs have been observed, with CD103^+^ DCs cross-presenting viral antigens in the mediastinal lymph node (MLN) at early time points of 2–4 days post-infection, as compared to CD11b^high^DCs at 5–7 days post-infection, possibly contributing to the development of different T cells subsets [30]. Moreover, the selective loss of CD103^+^ respiratory DCs reduces antigen-specific CD8^+^ T cell responses and only CD103^+^ DCs are capable of cross-presenting antigens in the lymph nodes [65], which suggests that CD103^+^ respiratory DCs contribute to the development of antiviral CD8^+^ T cell immunity.

To discriminate between memory precursor cells and effector T cells, killer-cell lectin-like receptor G1 (KLRG1) has been used in mice; short-lived effector CD8^+^ T cells are generated from KLRG1^high^ precursors, and all memory CD8^+^ T cells are from KLRG1^low^ precursors [66,67,68] (Figure 1). The TCR sequences used by these T cells showed an overlap between T_CM_ and T_RM,_ suggesting that they could derive from a single naïve T cell [69]. However, it is unclear whether an individual memory precursor has the potential to differentiate into both T_RM_ and T_CM_ cells [70]. 

The generation and establishment of T_RM_ cells in each tissue can significantly differ [71], indicating that tissue-specific environments play pivotal roles in these processes [72]. Moreover, the transcriptional regulation of these T cells poses specific requirements that vary between mice and humans [73,74]. However, both effector and memory T cells exhibit a similar pattern of chromatin accessibility, as compared to naïve CD8^+^ T cells [75]. Particularly, the transcription factors (TFs) Blimp-1, ID2, T-bet and STAT4 promote the development of T_EM_ [67,68,69,70,71,72,73,74,75,76,77,78,79], while the expression of BCL-6, Eomes, ID3, TCF-1 and STAT3 are associated with the generation of T_CM_ (Figure 1c, blue boxes) [80,81,82,83,84]. In mice, T_RM_ cells show an elevated expression level of the homolog of B lymphocyte-induced maturation protein-1 in T cells (Hobit), B lymphocyte-induced maturation protein-1 (Blimp-1) and Runx3 [85]. Together these TFs contribute to differentiation, residence and survival of T_RM_ cells in the tissue [86], and negatively regulate the expression of genes associated with tissue egress from non-lymphoid organs [87]. Nevertheless, at the protein level, Blimp-1 expression increased only during the effector stage, while Hobit mRNA and protein expression was constitutive during quiescence and downregulated after activation [88,89]. Both transcriptional factors Hobit and Blimp-1 have overlapping target genes that include cytotoxic molecules, such as granzyme B. During effector differentiation, CD8^+^ T cells acquire these cytotoxic molecules. Blimp-1 initiates the cytotoxic effector function because it is required for expression of granzyme B in murine effector T cells and T_RM_ cells, while Hobit maintains granzyme B expression in murine T_RM_ cells during their memory phase [88]. After influenza virus infection, CD8^+^ T_RM_ require continuous recruitment from the circulating memory pool to establish tissue resident cells, and this viral infection shows that Blimp-1, rather than Hobit, mediates the formation of T_RM_ cells within the lungs [86], potentially through control of the lineage choice between T_CM_ and T_RM_ cells during the differentiation of influenza-specific CD8^+^ T cells [87]. These data suggest that T_RM_ cell differentiation towards a unique program of transcriptional regulation occurs in a pathogen-specific manner. Besides, the differentiation of T_RM_ cells is highly dependent on local cues from the microenvironment and it has been shown that the expression of CD103 on T cells is dependent on TGF-β, which is required for T_RM_ cell establishment in some tissues. Indeed, the best-characterized signal required for T_RM_ cell development is mediated by TGF-β [90]; thus, CD8^+^ T cells that lack TGFβR fail to upregulate the expression of CD103 after antigen exposure and subsequently fail to differentiate into T_RM_ cells [89,90,91,92]. The downregulation of transcription factors KLF2, Eomes and the low expression of T-bet and TCF 1 is needed for proper T_RM_ cell residency [93]. Both T-bet and Eomes transcription factors control and down-regulate the signaling of TGF-β and upregulate CD103 [91]. Also, low levels of T-bet are required to maintain cell surface expression of the IL-15 receptor β-chain (CD122), which has been shown to be critical for the rapid proliferation of memory CD8^+^ T cells. In response to viral infection, type-I IFNs trigger the expression of IL-15 that prepares memory T cells for rapid division, independently of antigen re-exposure, by transiently inducing cell-cycle progression by the mTOR complex-1 (mTORC1) signaling pathway [92,94,95].

The type II C-lectin CD69 was initially related as an early T cell activation marker because its expression is rapidly induced on the surface of activated T cells [96]. Besides, CD69 is an antagonist of the sphingosine-1-phosphate receptor (S1PR1) that limits egress by blocking responsiveness to sphingosine-1-phosphate (SP1) gradients. The S1PR1 expression is commanded by the transcription factor Krüppel-like factor 2 (KLF2), and its downregulation is needed for T_RM_ cell formation in mice [97,98]. The constitutive expression of CD69 is essential for T_RM_ cell formation and is a typical marker of peripheral T_RM_ cells [93,98].

Another relevant surface molecule is CD44, a C-lectin that can act as a receptor for hyaluronic acid (HA). This molecule is maintained at high levels in T_RM_ cells populations, but CD44 alone does not single out T_RM_ cells from other CD8^+^ T cell populations [99]. The most relevant function of CD44 is the maintaining of cell structure through adherence to the extracellular matrix, mainly composed by HA [100,101]. 

The very late antigen 1 (VLA-1), also known as CD49a, is the α-subunit of the α_1_β_1_ integrin receptor that pairs with CD29 (integrin β_1_) [102]. CD49a is an adhesion molecule that binds to collagen IV (Coll IV) present in the basement membrane of epithelium [103]. Functionally, the blocking or genetic deletion of CD49a results in smaller T_RM_ cell population at the mucosal site, which becomes susceptible to secondary infections. These data suggest that CD49a plays a striking role in keeping these cells in the lungs [104,105].

CD103 is α-subunit of the integrin α_E_β_7_. It is involved in tissue retention by its interaction with the ligand E-cadherin and is upregulated on T_RM_ cells upon exposure to TGF-β [106]. In a mouse model of influenza infection, CD103 contributes to the retention of pathogen-specific cytotoxic T lymphocytes in the lungs and CD103 deficiency results in a reduction of CD8^+^ T_RM_ cells in the lungs [107,108]. Also, the expression of CD103 is essential for T_RM_ cell formation and constitutes a typical surface marker of T_RM_ cells.

T_RM_ cells in each tissue are maintained by different microenvironments, implicating that the process of the establishment and renewal of these cells varies between tissues (reviewed in [109] and [110]). This also implies that the signals and mechanisms of action are also dependent on the tissue milieu and, eventually, in the formation of particular structures for their maintenance [111,112,113]. For instance, in the lung tissue, an injury promotes the temporary generation of spatial niches called Repair-Associated Memory Depots (RAMDs) that contribute to tissue regeneration and constitute a niche that supports T_RM_ cell maintenance in the lungs [114]. Furthermore, the time for which T_RM_ cells persist in each tissue over time may vary. Several studies have shown that the dynamics of the establishment/renewal of T_RM_ cells in the lungs is different as compared to the female reproductive tract (FRT) or the skin [110]. Particularly, the establishment of T_RM_ cells in the FRT and skin are trigged by antigen-independent inflammation that recruits memory CD8^+^ T cells [115]. However, in the lung tissue, T_RM_ establishment depends on cognate-antigen and inflammation that promotes the formation of RAMDs [116]. On the other hand, the presence of memory T cells in the lung airways is maintained by continual recruitment from circulation [117], and presumably TRM cells in the airways could be supported by RAMDs. Nonetheless, the transient formation of RAMDs may account for the shorter longevity of CD8^+^ T_RM_ cells in the lung as compared to the gut and skin [22]. In addition, another essential aspect of T cells proliferation and differentiation is the exposure to damage/danger-associated molecular patterns (DAMPs), such as the nucleotides ATP and NAD^+^ [118]. CD8^+^ T_RM_ cells express the P2RX7 receptor, but not T_CM_ cells. Thereby, during infection or tissue damage, the release of DAMPs contributes to depleting T_RM_ locally [119]. For example, P2RX7 activation in vivo by exogenous NAD^+^ led to a specific depletion of T_RM_ while retaining circulating T cells, suggesting that the P2RX7 pathway contributes to the regulation of T_RM_ maintenance [120].

T_RM_ elicits a faster and more potent response after antigen recognition than circulating memory T cells [121]. T_RM_ triggers an innate-like alarm characterized by the production of massive amounts of effector molecules, such as: IFN-γ, TNF-α and granzyme B, as well as pro-inflammatory cytokines, chemokines and antimicrobial molecules [122]. The contribution of T_RM_ cells in mediating protective responses has attracted the attention for developing vaccination strategies that exploit the ability of these cells to establish long-lasting residency, which would prevent infections, particularly respiratory infectious diseases [22,23,24,25,26,27,28,29,30,31,32,33,34,35,36,37,38,39,40,41,42,43,44,45,46,47,48,49,50,51,52,53,54,55,56,57,58,59,60,61,62,63,64,65,66,67,68,69,70,71,72,73,74,75,76,77,78,79,80,81,82,83,84,85,86,87,88,89,90,91,92,93,94,95,96,97,98,99,100,101,102,103,104,105,106,107,108,109,110,111,112,113,114,115,116,117,118,119,120,121,122,123,124].

## 4. T_RM_ cells in Response to RSV Infection 

Both in mice and humans, natural infection with RSV induces the generation of virus-specific CD8^+^ T_RM_ cells that provide protection against RSV infection and contribute to reducing the severity of the disease. In the murine model, the adoptive transfer of airway CD8^+^ T_RM_ from RSV-infected mice into naïve recipients diminished the disease severity upon viral challenge. Additionally, RSV infection in mice induced T_RM_ recruitment in the airways and lung parenchyma [23]. Moreover, a study performed with humans showed that adults with higher frequencies of RSV-specific CD8^+^ T cells in the airways (many of which show a T_RM_ phenotype), developed less severe lower respiratory tract symptoms and reduced viral loads upon RSV challenge. It is noteworthy that increased protection against RSV did not correlate with an increase in circulating CD8^+^ T cells in the blood, but instead correlated with increased abundance of a preexisting T_RM_ cell population in the airways prior to infection [125]. Interestingly, during acute infection, RSV-specific CD8^+^ T_CM_ cells were temporarily accompanied by a CD103^+^/CD69^+^ population that could represent a cell population that migrates from the blood to the airways, which could represent T_RM_ cells [125].

In a non-human primate model, the infection of juvenile naïve African green monkeys with RSV showed that the peak of abundance of virus-specific CD8^+^ T cells emerging in peripheral blood and bronchoalveolar lavage (BAL) coincided with the declining of viral titers, suggesting that virus-specific cellular responses contribute to the clearance of RSV infection [21]. Moreover, this study showed an abundant CD103^+^ CD69^+^ population in BALs among the T cell population of these monkeys.

Influenza virus is another major respiratory pathogen, and CD8^+^ T_RM_ cells in nasal epithelia reduce viral transmission to the lungs, preventing pulmonary infection [126]. During secondary influenza virus infection, T_RM_ cells were shown to limit early viral replication, attenuating the duration of inflammation in an IFN-γ-dependent fashion. Additionally, specific T_RM_ showed an indispensable role in cross-protection against different influenza virus strains. These data suggest that T_RM_ plays a critical role in protection against respiratory infections in the upper and lower respiratory tracts [40,127].

In a mouse model of influenza and Sendai virus infection, the intratracheal transfer of antigen-specific CD8^+^ T_RM_ cells from immunized into naïve mice was sufficient to confer protection against respiratory virus infection. Interestingly, upon antigen exposure, the T_RM_ secreted IFN-γ quickly enough to limit viral replication in the lungs [128]. Additionally, the transfer of airway-resident IFN-γ-deficient CD8^+^ T_RM_ cells had a reduced protective potential. These findings suggest that airway-resident CD8^+^ T_RM_ cells contribute to the generation of protective immunity against respiratory pathogens and to the rapid secretion of IFN-γ, which is essential for achieving a successful viral clearance [128,129].

## 5. Tissue-Resident T Cells as a Target for Vaccine Development

While B cells mount responses against pathogens’ surface epitopes, CD8^+^ T cells attack mainly the internal epitopes of pathogens, which are more conserved and therefore prevent the pathogen’s escape of the immune system via antigenic drift. Therefore, the remarkable ability of CD8^+^ T_RM_ cells to serve as a first line of defense against reinfecting pathogens has become attractive so as to develop vaccination strategies that take advantage of the ability of T_RM_ cells to prevent respiratory infectious diseases [6,26,30]. Moreover, intranasal RSV infection in mice leads to the generation of antigen-specific CD8^+^ T_RM_ cells (CD103^+^/CD69^+^) in the lungs and airways. It has been shown that intranasal transfer of CD8^+^ T_RM_ cells from the airways of previously infected animals to naïve animals reduces weight loss upon infection. Additionally, the transfer of airway pan-CD8^+^ cells protected the body upon infection, ameliorating illness, reducing viral load and increasing IFN-γ secretion in the airways of the recipient mice [23].

Experimental RSV vaccines are capable of inducing protective memory T cells, such as the recombinant *Mycobacterium bovis* bacillus Calmette-Guèrin (BCG) expressing the RSV-N protein (rBCG-N-hRSV vaccine expressing RSV-nucleoprotein N), which is capable of eliciting protective immune responses [130,131]. This promising formulation developed under good manufacturing practices (GMP) has been shown to be safe in preclinical models and induces a strong antiviral T_H1_/T_H17_ T-cell memory response [132]. Additionally, some formulations are capable of generating T_RM_ cells. For example, intranasal administration of virus-like particles containing RSV M and M2 proteins has also been shown to induce T_RM_ cell development in the lung [133]. Moreover, DCs pulsed with *Listeria monocytogenes* as an immunization strategy were able to avoid the immunopathology caused by circulating T cells, and protect against a subsequent RSV challenge by promoting the generation of T_RM_ cells when administered locally [134]. 

The route of antigen delivery is relevant for gathering T_RM_ to the target tissue. For example, immunization with recombinant cytomegalovirus expressing the RSV M protein generated T_RM_ and T_EM_ CD8^+^ T cells populations when administered intranasally. However, when the vaccine was administered intraperitoneally, these cell populations were undetectable [21]. These findings support the notion that the route of antigen delivery in a vaccination context is a crucial determinant of immune priming at the infection site. Controversially, the immunization with a vaccine candidate G1F/M2 plus CpG as an adjuvant by the intraperitoneal route elicits high levels of T_CM_ cells and T_H1_ type of T_EM_ cells in the spleen, which may contribute to inhibition of lung inflammation. Nevertheless, the intranasal immunization recruits high levels of T_RM_ cells that lack or possess a weak T_H1_ type immune memory that transfers from the spleen into the lung, which might promote lung inflammation following RSV infection [135]. 

An interesting approach for generating T_RM_ cells is designated “prime and pull,” which combines vaccination (prime) with local administration of chemokines, adjuvants or antigens to recruit T_RM_ precursors to target tissues (pull) [136]. For example, zymosan can promote differentiation of effector CD8^+^ T cells to their differentiation in lung T_RM_ cells when administered as an adjuvant into the airways without any antigen [137]. 

As previously described, the formalin-inactivated RSV vaccine developed in the sixties (FI-RSV) caused vaccine-enhanced respiratory disease (ERD). Recently, it has been described that modulation of the immune response via TLR agonists can help to regulate the exacerbated immune response elicited by FI-RSV. TLR3 agonist CpG together with Notch receptor inhibitor L685458 markedly reduced FI-RSV-enhanced airway pathology symptoms, such as weight loss, lung inflammation and airway hyperresponsiveness. Moreover, the combined treatment promoted protective CD8^+^ T_RM_ cells in the lungs. Interestingly, none of the treatments by themselves alone reduced ERD. The proposed mechanism seems to involve the suppression of T_H17_ memory responses and the promotion of tissue-resident memory cells. Additionally, these results imply that the modulation of lung immune memory with adjuvants might be a good strategy to generate T_RM_ cells with antiviral properties [138]. 

The migration of antigen-specific T cells into the lungs, among other non-lymphoid tissues, is key for containing peripheral infections, and its modulation is an important strategy for the development of prophylactic tools [139]. The homing of T cells can be taught and would be a strategy for T_RM_ cell establishment after immunization. Tissue-associated DCs are relevant for imprinting the tropism of T cells during the priming phase. For example, T cell activation by intestinal DCs generates a gut-tropism because these cells are able to suppress in T cells the expression of skin-homing receptors once the T cell is activated [140]. Moreover, the synthesis of specific metabolites by DCs in each tissue contributes to particular T cell migration programs defined by DC priming [141]. Therefore, repeated intranasal immunizations, which promote the generation of T_RM_ cells through the development of a precursor cell pool, may be a promising strategy for the establishment of long-lasting lung T_RM_-mediated immunity against respiratory pathogens.

## 6. Concluding Remarks

In summary, CD8^+^ T_RM_ cells are noncirculating adaptive immune cells that are localized as sentinels at the physical barriers that are susceptible to the entry pathogens. Located at such places, they are key for the avoidance of reinfections by previously encountered microbial agents. Their placing at these hotspots of pathogen encounters provides rapid protection against viral infections. However, the signals that stabilize the T_RM_ population in the lung have yet to be identified. 

Considering that RSV is a respiratory viral pathogen that infects the respiratory epithelia in order to replicate, we consider that vaccination strategies against RSV should promote cell-based immunity that relies on the contribution of CD8^+^ T_RM_ response in the lungs. This claim is supported by the fact that CD8^+^ T cells in mice infected with RSV are essential for the clearance of this virus upon secondary infection. However, pre-existing RSV-specific memory CD8^+^ T cells migrating into the lungs lead to exacerbated disease severity and promote lethal immunopathology, mediated by the abundant secretion of IFN-γ in the lungs [142]. Therefore, CD8^+^ T cells are a critical subset that can positively contribute to viral resolution, or negatively contribute to vaccine-enhanced respiratory disease [134,143]. In addition, the establishment of a protective immune response against RSV requires neutralizing antibodies that reduce the infectivity of virions [144]. Thus, a cell-based immunity that recognizes virus-infected cells and neutralizing antibodies that prevent the propagation of virus is desirable in an effective vaccine. 

An attractive approach for generating T_RM_ cells was recently described and named “prime and pull,” which combines vaccination (prime) with the local administration of chemokines, adjuvants or antigens to recruit T_RM_ precursors that target particular tissues (pull) [136]. This strategy has the ability to recruit circulating effector T cells and memory T cells to sites where pathogens may enter, resulting in the generation of local T_RM_ that may significantly contribute to protection against viral infections caused by viruses, such as herpes simplex virus type 2 [18], malaria at the liver-stage of infection [145,146], human papillomavirus [147,148], influenza and even for developing cancer vaccines [149]. The main characteristics of these cells are their long permanence in the tissues, as well as their rapid recall responses that release molecules that mediate both antiviral and anti-tumor immunity. These attributes are desirable in the development of vaccines against viral pathogens and for cancer vaccines [150]. Thus, a detailed characterization of tissue-specific factors that regulate T_RM_ biology and their contribution both in pathology and protection could be crucial to new therapeutic designs.
